# Diminution of Public Health Agency Authorities Post-*Loper*

**DOI:** 10.1017/jme.2024.165

**Published:** 2024

**Authors:** James G. Hodge, Maxwell Lauzon

**Affiliations:** 1. SANDRA DAY O’CONNOR COLLEGE OF LAW, ARIZONA STATE UNIVERSITY, PHOENIX, AZ, USA

**Keywords:** Administrative Agencies, Powers, Authorities, Regulations, Deference, Supreme Court

## Abstract

In a new era of regulatory oversight, the US Supreme Court upended traditional *Chevron* deference to agency interpretations of ambiguous Congressional provisions in *Loper* in June 2024. Federal courts were instructed to make their own assessments of statutory authorities amid an onslaught of public health agency challenges surfacing nationally. Even so, SCOTUS may be eyeing further limits on agency powers despite clear and substantial repercussions for the health of the nation.

Consistent with the US Supreme Court’s recent penchant for bowling over major precedents, SCOTUS overruled longstanding *Chevron* deference this past June 2024 in *Loper Bright Enterprises v. Raimondo* ^1^ (*Loper*). Since 1984, *Chevron* deference has guided judicial evaluations of federal agency interpretations of ambiguous statutory provisions. So long as agency interpretations of Congressional language were “reasonable,” SCOTUS instructed courts to defer to the same.^2^ Determining the application and scope of *Chevron* deference was not always easy, but the doctrine offered a workable compromise between federal agency authorities and judicial oversight. That is, until the Court abandoned it in *Loper* citing Congressional language via the Administrative Procedures Act (APA) and separation of powers principles.^3^

As observed below, substantial concerns over federal public health administrative agency authorities have arisen immediately following *Loper.* ^4^ Compounding the effects of *Loper*, the Court in the same term expanded allowable time periods to sue administrative agencies^5^ and limited the jurisdiction of administrative law judges.^6^ Unsurprisingly, challenges of major agency determinations of health care and public health authorities are percolating. Entire areas of settled administrative law are subject to re-determination despite the Court’s assurances of the limited applications of *Loper*. Worse still, SCOTUS may not have reached its zenith in curtailing agency powers. Resulting instabilities underlying administrative agency rulemaking will assuredly impair the public’s health.

**Dissolution of *Chevron* Deference**. The facts of *Loper* (and its companion case^7^) are unremarkable. Innocuous provisions of the Magnuson-Stevens Fishery Conservation and Management Act^8^ empowered the federal National Marine Fisheries Service (Service) to require fishing vessels to defray costs of monitoring compliance with the Act. Lower courts applying *Chevron* deference agreed the Service had sufficient authority to mandate compliance. Whether these courts properly adjudged the legality of the Service’s rules under the Act was inconsequential. SCOTUS solely agreed to review the cases because the judges, like thousands of others over decades, relied on *Chevron* deference in making such determinations.

Characterizing *Chevron* deference as “unmoored,” “misguided,” and “unworkable,” among other adjectives, Chief Justice Roberts concluded for a 6–3 majority in *Loper* that federal courts “may not” defer outright to agencies’ interpretations of ambiguous statutes.^9^ Citing statutory language from the APA and broad separation of powers principles, the Court unequivocally abandoned *Chevron* deference after 40 years of applications. In its place, it offered new guidance for courts reviewing ambiguous statutory authorities for agency regulations. “Courts must exercise their independent judgment in deciding whether an agency has acted within its statutory authority….”^10^ While “[c]areful attention” to executive branch input may “help inform [a court’s] inquiry,”^11^ judges should not rely on agency interpretations in such cases. Even when Congress statutorily delegates federal agencies with express, specific powers, courts must assure agencies stay within their lanes.

The significance of the Court’s decision in *Loper* cannot be overstated. It rejected long-standing reliance on agency interpretations of ambiguous Congressional delegations in favor of courts’ independent judgements about the meaning of federal statutory laws. As Justice Kagan dissented in *Loper*, abandoning *Chevron* deference carries profound consequences. It “puts courts at the apex of the administrative process … because there are always gaps and ambiguities in regulatory statutes.”^12^ Unfortunately, as she observes further, courts are not well-positioned to ascertain legalities in cases involving specialized agency knowledge. Courts are essentially flying blind in assessing specific meanings of law in intricate areas of agency expertise and authorities.^13^

Unsurprisingly, challenges of major agency determinations of health care and public health authorities are percolating. Entire areas of settled administrative law are subject to re-determination despite the Court’s assurances of the limited applications of Loper. Worse still, SCOTUS may not have reached its zenith in curtailing agency powers. Resulting instabilities underlying administrative agency rulemaking will assuredly impair the public’s health.

***Loper* Effects on Administrative Agency Authorities.** *Loper’s* impacts on health agencies are profound and troubling. Agencies were already dealing with restrictions on their authorities via the Court’s enunciation of its “major questions doctrine” in 2022, severely questioning administrative rules in areas of “economic or political significance.”^14^ Since *Loper*, litigants have already objected to key agency regulations. These include challenges concerning: (1) the ability of the Department of Health and Human Services (HHS) to suspend Medicare payments against providers facing credible allegations of fraud;^15^ (2) Food and Drug Administration’s (FDA) authority to regulate “biological products” via the Public Health Service Act;^16^ (3) the breadth of HHS’s power to exclude individuals from federal healthcare programs after convictions for fraud under the Social Security Act;^17^ and (4) enforcement mechanisms under the federal “Stark law” prohibiting physician referrals.^18^ Additional claims are even more ominous. In two companion cases on SCOTUS’ shadow docket,^19^ the federal Department of Education sought to temporarily enforce its April 2024 regulations issued pursuant to Title IX of the Education Amendments of 1972.^20^ These include several provisions protecting transgender persons from school-based discrimination. The Court refused to allow enforcement of the regulations while lower courts litigate their constitutionality. Ultimate legal resolution of these cases may rest in part on *Loper-*like assessments of agency authorities. Similar challenges to provisions of the Affordable Care Act and other seminal health laws are simmering nationally.

While *Loper te*chnically only applies to federal agencies, multiple state courts mimic SCOTUS’ determinations. Prior to *Loper*, nine states (AZ, DE, FL, KS, MI, MS, UT, WI, and WY) had already rejected *Chevron*-like deference concerning their own state agencies.^21^ Post-*Loper*, additional states have begun applying *Loper-*like principles.^22^ On July 17, 2024, the South Carolina Court of Appeals reversed a lower court’s granting of a pollution control tax exemption, reasoning that the lower court did not correctly assess the state agency’s interpretation of the statutory term, “industrial plant.”^23^ In mid-August, a Georgia Court of Appeals judge called for the state’s supreme court to “drive a *Loper-*like stake in the heart of what remains of *Chevron*-style deference here in Georgia.”^24^

Not all state courts have followed suit.^25^ In August 2024, a Connecticut state trial court concluded that *Loper* “does not necessarily mean that *Chevron* deference no longer applies in state court.”^26^ On September 24, the Supreme Court of Hawaii echoed Justice Kagan’s concerns, observing that “*Chevron* made for good, balanced governance,” where courts “defer to those agencies with the na’auao (knowledge/wisdom) on particular subject matters to get complex issues right.”^27^ Without deference to agencies, judges must become experts on “exceedingly complicated areas of American life,” noted the court, including core public health issues like air quality, food and drug safety, and work-related wellness.^28^

Irrespective of state court express adaptations of *Loper’s* reasoning are the effects of shifting interpretations of federal public health agency rules directly relied on by state agencies as well. Multiple state legislatures and health agencies incorporate by reference express provisions of federal regulatory laws into their own state laws. Consequential changes to federal regulations via federal court interpretations reverberate across states as well. Repercussions intensify when federal courts diverge as to their interpretations, seek to assess seminal federal regulations like the HIPAA Privacy Rule^29^ or Common Rule,^30^ or alter emergency rules authorizing real-time options (as per extensive federal regulations relied on during the COVID-19 pandemic). Navigating federal regulatory requirements in major emergencies or catastrophes was already complex. *Loper* obfuscates these efforts further.

In the deeply politicized field of public health, shifting greater control to federal courts stocked with politically appointed, tenured judges may inhibit communal health advances for decades ahead absent affirmative Congressional interventions.

**Zenith of Limits to Administrative Rulemaking.** Intimating that SCOTUS has reached the pinnacle of its jurisprudence limiting agency authorities underestimates the conservative reach of the Court. In *Loper, s*ome Justices forecasted their willingness to reexamine what is known as *Auer* deference. As per its 1997 decision, *Auer v. Robbins*,^31^ Justice Scalia determined for a unanimous Court that it was reasonable for courts to defer to federal agencies’ determinations of the meanings of the agencies’ own rules. *Auer* deference is distinct from *Chevron* deference in that the former does not involve Congressional statutory ambiguities. This distinction may soon be called into question. As Justice Gorsuch suggested in his concurring opinion, “All *[Loper]* means is that, going forward, federal courts will … resolve cases and controversies without any systematic bias in the government’s favor.”^32^ To the extent that *Auer* deference, like *Chevron*, implicates a level of governmental “bias,” it seems poised for SCOTUS’ reexamination. Pending environmental and firearm cases before the Court this term present ample opportunities for the same. Notably, however, SCOTUS denied review of a Fifth Circuit decision upholding the independence of the Consumer Product Safety Commission on October 21, 2024.^33^ The case “tee[d] up one of the fiercest (and oldest) fights in administrative law” — specifically for-cause removal protections to members of quasi-legislative and -judicial federal commissions.^34^

The potential demise of *Auer* deference would plunge administrative regulatory authorities into unknown depths. If courts can instruct federal or state agencies on what the agencies’ own regulations mean without deference to agencies themselves, public health regulatory authorities may be abdicated entirely via adjudication. The driving premise of court assessments of agency regulations may no longer be the extent of their constitutional or statutory infirmity, but rather courts’ independent assessment of what agencies are — or should be — authorized to regulate. In the deeply politicized field of public health, shifting greater control to federal courts stocked with politically appointed, tenured judges may inhibit communal health advances for decades ahead absent affirmative Congressional interventions.
